# AID-U-Net: An Innovative Deep Convolutional Architecture for Semantic Segmentation of Biomedical Images

**DOI:** 10.3390/diagnostics12122952

**Published:** 2022-11-25

**Authors:** Ashkan Tashk, Jürgen Herp, Thomas Bjørsum-Meyer, Anastasios Koulaouzidis, Esmaeil S. Nadimi

**Affiliations:** 1 Applied AI and Data Science (AID), Mærsk McKinney Møller Institute (MMMI), University of Southern Denmark, 5230 Odense, Denmark; 2 Danish Center for Clinical AI (CAI-X), 5230 Odense, Denmark; 3 Department of Surgery, Odense University Hospital, 5000 Odense, Denmark

**Keywords:** biomedical images, convolutional neural networks, semantic segmentation, up and downsampling

## Abstract

Semantic segmentation of biomedical images found its niche in screening and diagnostic applications. Recent methods based on deep learning convolutional neural networks have been very effective, since they are readily adaptive to biomedical applications and outperform other competitive segmentation methods. Inspired by the U-Net, we designed a deep learning network with an innovative architecture, hereafter referred to as AID-U-Net. Our network consists of direct contracting and expansive paths, as well as a distinguishing feature of containing sub-contracting and sub-expansive paths. The implementation results on seven totally different databases of medical images demonstrated that our proposed network outperforms the state-of-the-art solutions with no specific pre-trained backbones for both 2D and 3D biomedical image segmentation tasks. Furthermore, we showed that AID-U-Net dramatically reduces time inference and computational complexity in terms of the number of learnable parameters. The results further show that the proposed AID-U-Net can segment different medical objects, achieving an improved 2D F_1_-score and 3D mean BF-score of 3.82% and 2.99%, respectively.

## 1. Introduction

Medical image segmentation based on deep learning convolutional neural networks (DL-CNN) has become a research hotspot. Semantic segmentation found its niche in diagnostic applications, resulting in lessening instances of invasive surgical procedures [1]. Applications of this technique include, but are not limited to, detection of bacterial objectives in microscopic slides, cellular lesions such as gastrointestinal polyps, and brain and prostate tumors.

In recent years, several studies have been conducted to provide reliable and robust AI-based models for semantic segmentation; however, the proposed methods suffer from various shortcomings such as sharp decrease in performance when a new objective target is introduced [2,3,4]. To overcome such issues, improvements such as interchanging from series convolutional neural networks (CNNs) to dilated acyclic graph CNNs, e.g., googleNet [5], ResNets [6] or DenseNets [7], have been implemented. In [8] and [9], the authors employed a combination of deep CNNs with a fully connected conditional random field (CRF) to overcome the challenge of dealing with weakly labeled data in CNNs. Another CNN-based method is known as fully convolutional network (FCN) [10,11,12,13,14,15,16,17,18]. FCN deploys a pixel-to-pixel mapping strategy, instead of region proposal extraction. The backbone of an FCN is usually a pre-trained CNN such as VGG16. The idea behind FCN is what became the backbone of U-Nets [19]. The fully convolutional DenseNet is another approach [17]. This approach improves the performance of the network for semantic segmentation, especially in upsampling or expansive paths.

The existing problem with all the previously proposed U-Nets is that their performance decreases as the complexity of the target objects for segmentation increases. The examples of such complexity for target objects are their complicated morphology, the high number of simultaneous samples and the complexity of the image modalities. Moreover, some of the proposed networks such as Circle-U-Net [20] have a fixed number of layers, preventing them to provide their best performance for segmenting different target objects.

In this paper, an innovative semantic segmentation method for pixel-wise classification of images, hereafter referred to as AID-U-Net, with a novel architecture is proposed. The architecture is inspired by U-Net, but our proposal outperforms and overcomes deficiencies and complexities of U-Net and other semantic segmentation methods. We applied AID-U-Net to various biomedical image databases of different quality, to demonstrate its enhanced efficiency in terms of both feasibility and performance criteria. The proposed network can be employed for training datasets that include a low number of images based on the structure of its architecture, i.e., the architecture can be reformed according to the resolution and number of dataset images. AID-U-Net is a fully flexible CNN for semantic segmentation applications capable of discriminating the foreground from background pixels. The main contribution of this work is two-fold:

- **Low Computational Complexity:** A lower number of learnable parameters with the same number of layers as those of a conventional U-Net, resulting in a faster learning convergence.

To justify the lower computational complexity of the proposed AID-U-Net compared with U-Net, the following descriptions can be presented:

If there are N regular blocks in each contracting and expansive path of a conventional U-Net, the computational complexity for such a network can be calculated as the summation of a geometric sequence of 2*^i^*s for the learnable parameters of all 2D convolutional layers in a conventional U-Net. The formula for calculating the sum of a geometric sequence is as follows:

(1) $$\overset{N}{\underset{n=0}{\sum }}a_{1}r^{n}=a_{1}\frac{r^{N+1}-1}{r-1}$$

where $a_{1}$ is the first term and *r* is the common ratio of the geometric sequence.

When *r* = 2 and $a_{1}$ = 1, the summation of such a geometric sequence would be equal to 2*^N^*^+1^ − 1. Thus, the worst order for computational complexity of a U-Net with a depth of N for each contracting or expansive paths would be O(2*^N^*^+1^ − 1).

On the other hand, there are direct paths with the depth of K and sub-paths with the depth of d in a proposed AID-U-Net, such that *K* + *d* = *N*. The worst computational complexity for such architecture can be calculated as follows:

(2) $$S_{1}=\overset{K}{\underset{n_{1}=0}{\sum }}2^{n_{1}}=\left(\frac{2^{K+1}-1}{2-1}\right)=2^{K+1}-1$$

(3) $$S_{2}=\overset{K-d}{\underset{n_{2}=K}{\sum }}2^{n_{2}}=-\left(\frac{2^{K-d}-2^{K+1}}{2-1}\right)=2^{K+1}-2^{K-d}$$

where *S*_1_ is the summation of a geometric sequence in the direct path of a sample AID-U-Net with length *K*, and *S*_2_ is the summation of a geometric sequence in the sub-path of a sample AID-U-Net with length d. Given that, the worst computational complexity for an AID-U-Net with a depth of *K* for direct-path and a depth of d for the sub-path can be calculated as O($S_{1}+S_{2}$) = O($2^{K+2}-2^{K-d}-1$). It can be readily shown that O(2^*N*+1^ − 1) is larger than O($2^{K+2}-2^{K-d}-1$) under the conditions *N* = *K* + *d*, and *d* ≤ *K*. For instance, if *N* = 5 and *K*, *d* = 3, 2; the order of computational complexity for U-Net (5) and AID-U-Net (3, 2) that has the same number of deep convolutional layers would be O(63) and O(29), respectively.

- **Dimensional Flexibility:** AID-U-Net’s architecture is designed to segment target objects in both 2D and 3D, by counting for both depth and width of the input data (unlike ResNet-based networks).

In the proposed strategy, we deployed a specific approach for the preparation of the input images before feeding them into the deep neural network. Based on this approach, the input images with any dimensionality and resolution are partitioned into smaller sub-images known as patches, where the size of the input layer for the proposed AID-U-Net is equal to the size of the patches. Before extracting the patches out of the original images, the images should be cropped. The cropping procedure alongside extracting the patches with constant sizes organize the dimensional flexibility. The details of the proposed dimensional flexibility based on the cropping procedure and patch extraction are proposed under the later sub-sections entitled pre-processing and cropping procedure. 

In the real world, it is essential to overcome the hardware limitations for the purpose of real-time object detection. Therefore, the importance of lowering the computational complexity by reducing the number of learnable parameters and providing more dimensional flexibility by preserving the original resolution of the imaging modality play important roles in practical applications such as the one proposed in [21].

## 2. Literature Review

**Figure 1:**
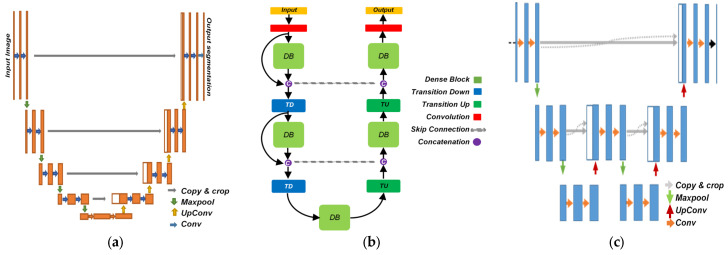
Graphical comparison between (**a**) U-Net [19], (**b**) fully convolutional DenseNet [17], and (**c**) proposed AID-U-Net.

There are a plenty of semantic segmentation networks that have a similar architecture as U-Net. It is possible to distinguish them from each other based on the schematic of their architectures. A graphical comparison between the architecture of a conventional U-Net, a fully convolutional DenseNet, and the proposed AID-U-Net is shown in Figure 1. Other semantic segmentation methods are either derived from or are similar to U-Net, such as Feed-forward FCNs such as Fully Convolutional DenseNet [22], DeepLabv3+ [23] and FastFCN [24], V-Nets [25] for semantic segmentation of 3D volumetric images, W-Net [26] as a mostly unsupervised segmentation strategy, and ResU-Net [27] with a revised version of sampling blocks based on the residual concept.

**Figure 2:**
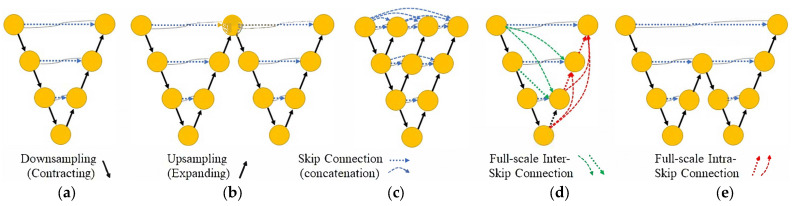
Graphical visualization of different architectures for both the existing state-of-the-art U-Net models and the proposed AID-U-Net with a direct path of length 4: (**a**) Wide or Vanilla U-Net, (**b**) W-Net (Series or Cascaded U-Net), (**c**) U-Net++ (Nested U-Net), (**d**) U-Net3+ (Full-scale skip), and (**e**) AID-U-Net.

Another approach toward the improvement of automatic segmentation of medical images based on DNNs with a U-Net backbone is proposed in [28], also known as no New U-Net (nnU-Net). The main idea behind nnU-Net is that proper pre-processing, hyper-parameter selection and implementation of the training procedure play a crucial role in image segmentation. However, this statement that the choice of these configurations is often more important than the actual network architecture has been questioned. Networks such as UNet++ [29,30,31,32,33,34] and UNet3+ [35] have proven that applying modifications to the architecture of the baseline network U-Net [14] resulted in better segmentation outcomes. These methods deploy architectural innovations that provide better segmentation performance, but at the cost of increasing structural and computational complexities. For instance, the difference between the efficiency of UNet++ and UNet3+ applied to the same database of images is just about 1.4%; however, the number of learnable parameters for the same input layer dimensionality is significant. Figure 2 presents a graphical illustration of different semantic segmentation networks and their architectural comparison.

Table 1 provides a short overview of the most recent state-of-the-art CNN methods for semantic segmentation purposes targeted at biomedical imaging.

## 3. Methods

**Figure 3:**
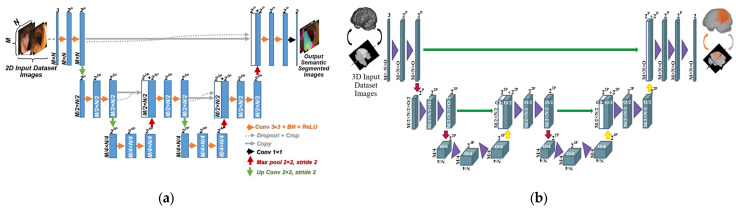
(**a**) Two-dimensional AID-U-Net architecture with Direct path depth 2 and sub-path depth 1; (**b**) three-dimensional AID-U-Net architecture with direct path of depth 2 and sub-path of depth 1.

The architectural structure of the 2D and 3D versions of AID-U-Net is illustrated in Figure 3a,b, respectively. To allow for a seamless tiling of the output segmentation map, all 2 × 2 max-pooling operations are applied to a layer with even *x* and *y* axes.

The architecture resembles U-Net from the perspective of fundamental CNN components such as, up- and downsampling convolutions, ReLUs, max-pooling, concatenations, sigmoid activation function, batch normalization and dropouts. However, the following distinguishing key features need to be highlighted:

- Cropping procedure that makes the current CNN model applicable to different image sizes.
- Modified classification layer with generalized dice loss function to suit various types of input data and image qualities.
- Significant reduction in computational complexity in terms of the number of learnable parameters, compared to that of a U-Net with the same number of layers.

As presented in Figure 3, several contracting and expansive paths are deployed in AID-U-Net. A one-sample AID-U-Net has one main contracting path with the possibility of further upsampling path, as well as one main upsampling path followed by the same number of sub-contracting paths.

### 3.1. Deep Layers Combinations and Block Formation in AID-U-Net

AID-U-Net is composed of two main paths: Contracting path that leads to downsampling the input images into feature vectors (matrices), and expansive path that joins the related connected components to each other, resulting in a semantic segmentation output. In addition to these two distinct paths, there are two main building blocks for each path as follows:

- **In Contracting path:** A combination of cascaded convolution, Batch normalization, ReLU, convolution, Batch normalization, ReLU, dropout and cropping layer form a block called contracting block.
- **In expansive path:** A combination of cascaded concatenation, Batch normalization, ReLU, convolution, Batch normalization, ReLU and dropout layers form another block called expansive block.

The convolutions are padded; otherwise, max pooling of the unpadded convolutions leads to improper shape blocks. The connection between consecutive contracting blocks is established by a max-pooling layer to reduce dimensionality and to select maximal discriminating features.

#### 3.1.1. Pre-Processing

To overcome the issue of underfitting in deep convolutional neural networks due to the inadequate number of input images, it is necessary to augment the images existing in the datasets. The deployed augmentation procedures are rotation, translation, and reflection. Moreover, for making more samples out of the dataset images based on their original dimensions to prevent loss of information because of shrinking and squeezing, the images are divided into sub-images known as patches.

The number and size of the extracted patches are in association with the size of the input layer for the proposed AID-U-Net model and the initial size of the input images. For instance, if the assigned input dimension for the network is 256 × 256 × 3, and the sizes of the images in the dataset are 256 × 512 × 3, then there would be two patches for each input image plus the number of augmented versions extracted for each patch. If the initial dimension of the images is not divisible by the patch sizes, then an overlapping between the patches is inevitable.

#### 3.1.2. Cropping Procedure

**Figure 4:**
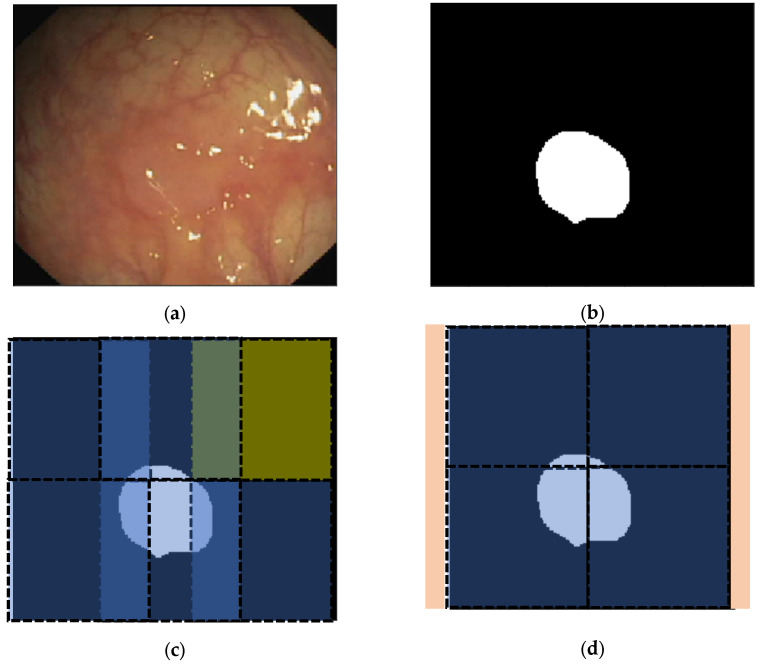
(**a**) A sample CVC-ColonDB image with original size of 574 × 500 pixels; (**b**) the ground truth image for the polyp as the target object, (**c**) extraction of patches with the size of 256 × 256 (the yellow patch does not include any part of the polyp as the ground truth), and (**d**) patch extraction after applying appropriate cropping procedure to the original image (there is no patch without the presence of the polyp as the target object).

As extraction of patches may lead to losing details or to creating redundancies, especially in the case of overlapping of the patches, the cropping procedure helps us avoid such issues. When there are several target objects in the same input image, then cropping alongside patch extraction will prevent patches that have lost a part or the whole of a target object, and there would not be two completely similar target objects in two or more than two patches. Cropping is not necessary for the cases in which there is no overlap between the extracted patches. The other point is that the cropping size should be adjusted for each dataset separately. A sample procedure for visualizing the effective role of cropping is depicted in Figure 4. 

### 3.2. Functionality of Downsampling (Contracting) and Upsampling (Expansive) Blocks

An up-convolutional layer connects consecutive expansive blocks to each other, providing upsamples of local features, and injecting those outcomes toward the output blocks. The role of concatenation layers in the expansive blocks is to make a compromise between local and global features from the same blocking rows. By concatenating the output content of two layers instead of adding them to each other within the expansive path, the variance of the output of each block will be the same as those of input layers:

(4) $$\sigma_{output}^{2}=\sigma_{input_{1}||input_{2}}^{2}=\sigma_{input_{x}}^{2}$$

where *x* stands for the index of each input content to the concatenation layer (||). The output of an addition layer, however, would have a variance two times larger than that of each input to that layer.

The output of AID-U-Net comprises two distinct layers. The first layer is a SoftMax layer, acting as an average pooling layer to unify the global features and form a final semantic segmentation result. The second consecutive layer with a sigmoid activation function is the classification layer that provides the outcome.

Each contracting block consists of the repeated application of two convolutions (usually *N* filters with the size of 3 × 3 with the same padding structure). The initial convolution is always followed by a batch normalization layer. The batch normalization layer provides a remarkable coordination for the outputs of the convolutional layers, especially in feedforward pass, and it is optimized in a backward learning pass [37]. It standardizes the activation of each input variable, especially for each of the deployed mini-batches, and supports higher learning rates [38]. Characterized by *γ* and *β* parameters, the standardized output *y_i_* over each mini batch is calculated as follows:

(5) $$y_{i}=BN_{\gamma ,\;\;\beta }\left(x_{i}\right)=\gamma \hat{x_{i}}+\beta$$

where both *γ* and *β* are updated during backpropagation training passes.

The two choices for the classification layer loss function, namely cross-entropy vs. dice coefficient, has been considered, in which the latter was preferred. This was due to the class imbalance in biomedical images, as some segments occupy less pixels/voxels than others [24]. The generalized dice loss function *L* for the loss between the predicted *P* and the corresponding ground truth *G* is given by:

(6) $$L=1-\frac{2\sum_{i=1}^{M}\omega_{i}\sum_{j=1}^{N}P_{ij}G_{ij}}{\sum_{i=1}^{M}\omega_{i}\sum_{j=1}^{N}\left(P_{ij}^{2}+G_{ij}^{2}\right)}$$

where *M* and *N* are the number of classes and the number of elements along the first two dimensions of *P*, respectively. $\omega_{i}$s stand for class contribution controlling weights. These weighting factors are the inverse area of the expected region for target objects and are computed as follows:

(7) $$\omega_{i}=\frac{1}{{\left(\sum_{j=1}^{N}G_{ij}\right)}^{2}}$$

Beyond the architectural sub-contracting and sub-expansive blocks, each block comprises batch normalization, dropout and cropping layers.

## 4. Implementation and Results

We tested two architectures of AID-U-Net featuring different depths of contracting/expansive and sub-contracting/sub-expansive paths on a set of distinct databases (Table 2), including both 2D and 3D volumetric images. The two AID-U-Nets constitute, respectively, a depth of 3 and 2 direct contracting/expansive, and 1 and 2 sub-contracting/sub-expansive paths, hereafter referred to as AID-U-Net(3, 1) and AID-U-Net(2, 2). The code is also available at https://github.com/ashkantashk/AID-U-Net (accessed on 22 October 2022).

### 4.1. Datasets

To compare the performance of AID-U-Net to its counterparts, we applied the standard U-Net to all the examples within the databases of Table 2. To compensate for a small training set, both patch extraction (splitting large size input data into smaller ones) accompanied by augmentation (scaling, translating, and rotating extracted patches) were carried out. The analyses were performed using a single NVIDIA GeForce RTX 2080 GPU with 8 GB dedicated memory.

### 4.2. 2D Optical Colonoscopy Images

**Figure 5:**
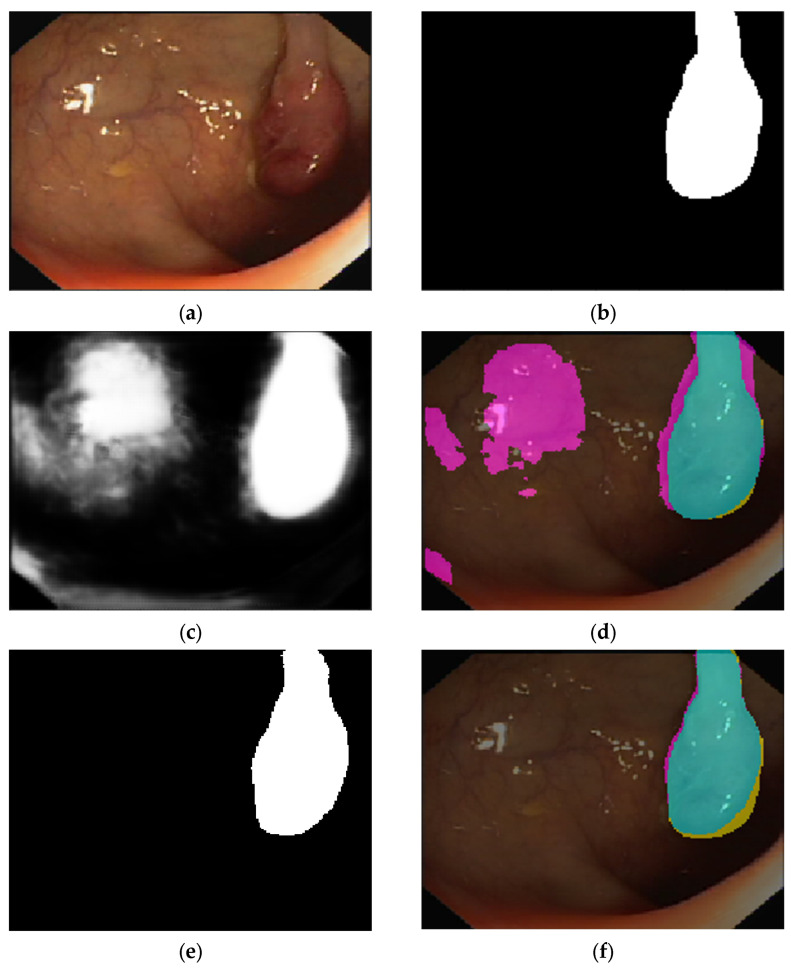

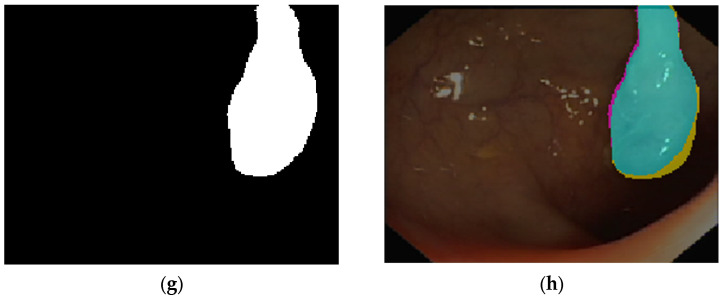
Sample confusion matrix visualization of sample colonoscopy image for: (**a**) test polyp and (**b**) its related ground truth, (**c**) extracted polyp, (**d**) confusion matrix overlay mask extracted by U-Net(4), (**e**) extracted polyp, (**f**) confusion matrix overlay mask extracted by AID-U-Net(3, 1), (**g**) extracted polyp, and (**h**) confusion matrix overlay mask extracted by AID-U-Net(2, 2) (■ (cyan): as TP pixels, ■ (magenta): as FP pixels, ■ (yellow): as FNs, and ■ (black) as TNs).

The first test bed to evaluate the performance of the AID-U-Net is the publicly available CVC-clinicDB database, featuring annotated images of colorectal polyps retrieved from optical colonoscopy (OC) [39,40,41]. A sample OC image featuring a colorectal polyp and the pixel-based ground truth are presented in Figure 5a,b, respectively. The final semantic segmentation and the visual confusion matrix for the U-Net of depth 4 are illustrated in Figure 5c,d, respectively. Moreover, the performance of AID-U-Net(3, 1) and AID-U-Net(2, 2) are presented in Figure 5e–h.

**Figure 6:**
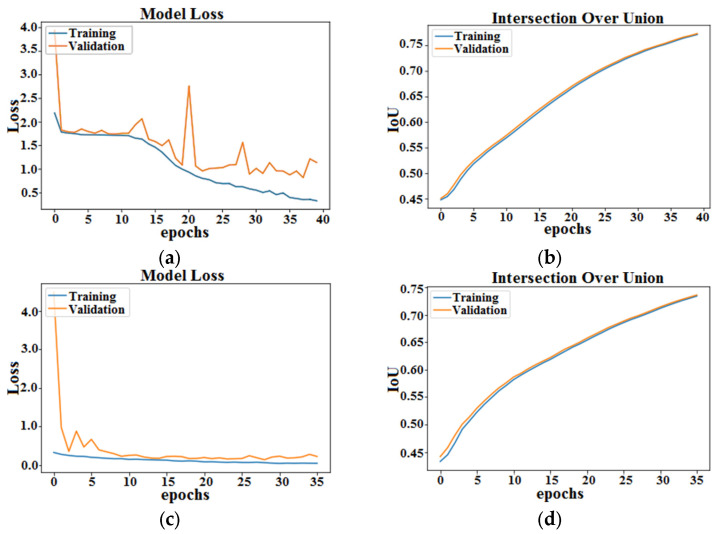

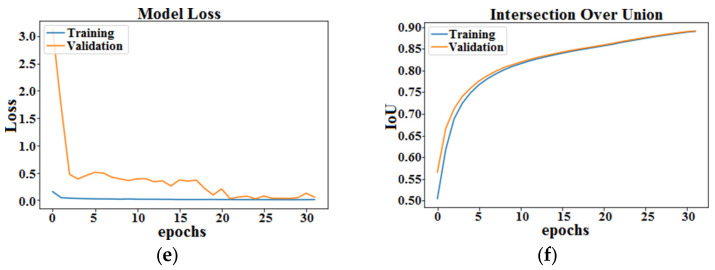
Summarized history for: (**a**) Loss of U-Net, and (**b**) Intersection over Union (IoU) of U-Net (**c**) Loss of AID-U-Net (3, 1), and (**d**) IoU of AID-U-Net (3, 1), (**e**) Loss of AID-U-Net (2, 2), and (**f**) IoU of AID-U-Net (2, 2).

Model loss and accuracy per epochs for both AID-U-Net (3, 1) and AID-U-Net (2, 2) are presented in Figure 6. As seen, the convergence criteria for AID-U-Net (2, 2) are met faster than that of AID-U-Net (3, 1), as the total number of parameters of AID-U-Net(2, 2) is significantly lower than that of AID-U-Net(3, 1). 

To quantify the efficiency of AID-U-Net in segmenting images, we further calculated the precision, recall, sensitivity, specificity, and accuracy, reported in Table 3. It is evident that the highest F_1_-score and IoU were achieved by AID-U-Net(3, 1), surpassing both U-Net++ and U-Net(4), while the lowest number of learnable parameters belonged to AID-U-Net(2, 2).

### 4.3. 2D Colon Capsule Endoscopy (CCE) Images

**Figure 7:**
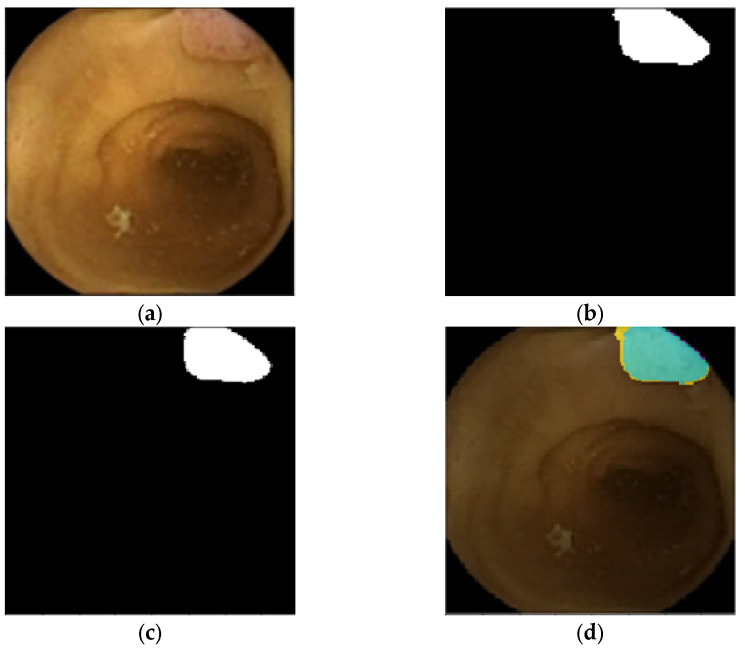

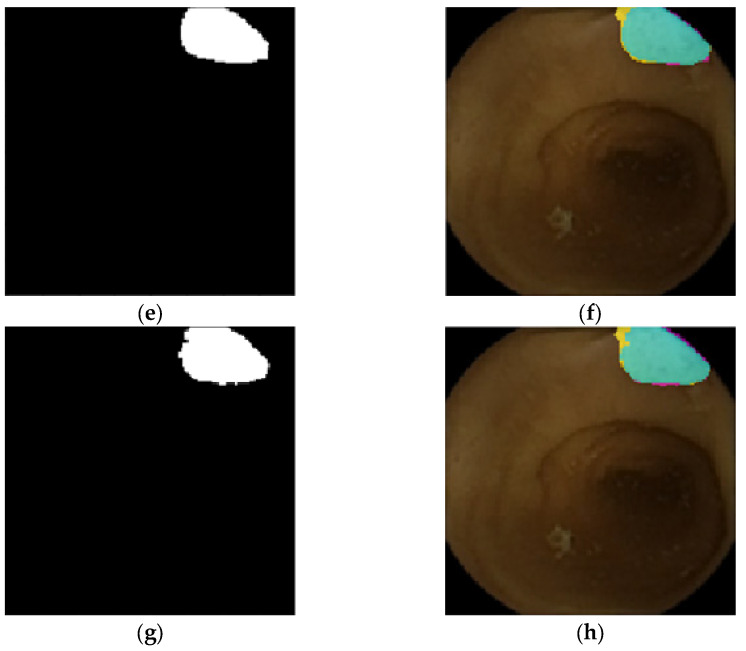
Sample confusion matrix visualization of sample CCE image: (**a**) Test polyp and (**b**) its related ground truth, (**c**) extracted polyp, (**d**) confusion matrix overlay mask extracted by U-Net(4), (**e**) extracted polyp, (**f**) confusion matrix overlay mask extracted by AID-U-Net(3, 1), (**g**) extracted polyp, and (**h**) confusion matrix overlay mask extracted by AID-U-Net(2, 2) (■ (cyan): as TP pixels, ■ (magenta): as FP pixels, ■ (yellow): as FNs, and ■ (black) as TNs).

A sample CCE image featuring a colorectal polyp and the pixel-based ground truth are presented in Figure 7a,b, respectively. The final semantic segmentation and the visual confusion matrix derived from a U-Net of depth 4, AID-U-Net(3, 1) and AID-U-Net(2, 2) are illustrated in Figure 7c–h.

The comparative results of the conventional U-Net and our AID-U-Net with filtering selection rate of 2*^N^* are reported in Table 4. Unlike the previous example, AID-U-Net(2, 2) achieves the highest IoU and F_1_-Score with the lowest number of learnable parameters.

In [45], we deployed AID-U-Net with different backbones for semantic segmentation of the CCE image dataset that were annotated by clinicians at Odense University Hospital (OUH). The comparative results demonstrate the superiority of the proposed model over the other state-of-the-art U-Nets even with pre-trained backbones.

### 4.4. 2D Microscopic Images

**Figure 8:**
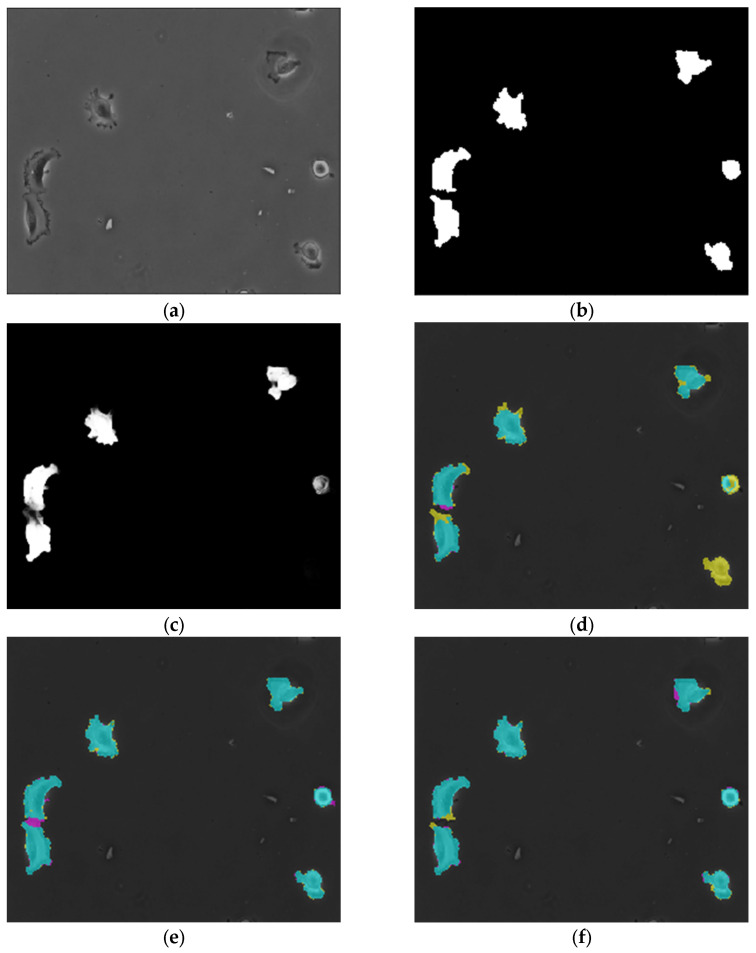
Comparison of objective confusion matrix results for sample M.O. detection: (**a**) original test M.O.s and (**b**) ground truth, (**c**) extracted M.O.s for U-Net(4), (**d**) confusion matrix overlay mask for U-Net(4), (**e**) confusion matrix overlay mask for AID-U-Net (3, 1), and (**f**) confusion matrix overlay mask for AID-U-Net (2, 2) (■ (cyan): as TP pixels, ■ (magenta): as FP pixels, ■ (yellow): as FNs, and ■ (black) as TNs).

In this example, a dataset containing 2D optical microscopy images with target objects being Glioblastoma-astrocytoma cells are deployed. This dataset was first introduced during the ISBI cell tracking challenge [42]. The results of the semantic segmentation obtained by a U-Net of depth 4, AID-U-Net(3, 1) and AID-U-Net(2, 2) are illustrated in Figure 8.

The results (Table 5) demonstrate that AID-U-Net (2, 2) outperforms all other networks within all evaluation metrics.

### 4.5. 3D Brain and Prostate Images

In this section, volumetric CT scan images of brain tumors and the prostate gland are used as the test bed [44]. The brain CT-scans feature approximately 400 brain voxels including annotated tumors. Each volume comprises 152 slices, and each slice is of size 176 × 224 × 4, leading to a volume size of 176 × 224 × 152 × 4.

Parameter configuration for the implementation of U-Net and AID-U-Net is provided in Table 6, showing that having the same number of layers, AID-U-Net is less complex than U-Net. This is because the number of learnable parameters in AID-U-Net is significantly lower, which in turn results in higher efficiency, faster training, and lower computational load (Table 6).

**Figure 9:**
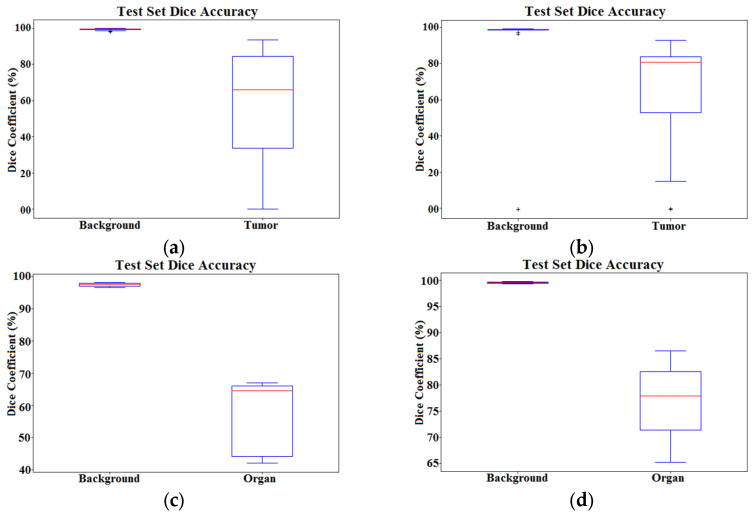
Boxplots for comparing between the efficiency of (**a**) conventional U-Net(3) and (**b**) AID-U-Net (2, 1), applied to brain CT scans for detecting and localizing brain tumors. Boxplots for comparing between the efficiency of (**c**) conventional U-Net (3) and (**d**) proposed AID-U-Net (2, 1), applied to 3D abdomen CT scans for detecting and localizing the prostate gland.

Dice score statistics across the volumes of both brain and abdominal CT scans to detect tumors and the prostate gland, respectively, are presented in Figure 9. 

The red lines in the plot present the median dice value for the classes, while upper and lower bounds of blue boxes indicate the 25th and 75th percentiles, respectively. Black whiskers extend to the most extreme data points that are not outliers. Head-to-head comparison of the performance of 3D U-Net(3) and AID-U-Net (2,1) for semantic segmentation of brain tumors in 3D brain CT scan voxels is presented in Table 7. 

In addition, head-to-head comparison of the performance of 3D U-Net(3) and AID-U-Net (2,1) for semantic segmentation of prostate gland within 3D abdomen CT scans is presented in Table 8.

It is evident that AID-U-Net(2, 1) outperforms 3D U-Net(3) in accuracy, mean and weighted IoU and BF scores. We further compared the performance of U-Net(5), V-Net(5) and AID-U-Net(3, 2) on the brain tumor image database, as presented in Table 9.

Even though AID-U-Net(3, 2) does not outperform its counterparts in global and mean segmentation accuracy, its performance qualifies it as the best performing network, owing to a large margin of improvement over IoU and BF scores, and most importantly, the significant reduction in the number of learnable parameters compared to those of U-Net and V-Net. Furthermore, it is evident that the difference in performance over global and mean accuracy is negligible, scoring only 0.8 and 1.8% lower than the highest scoring networks, respectively.

**Figure 10:**
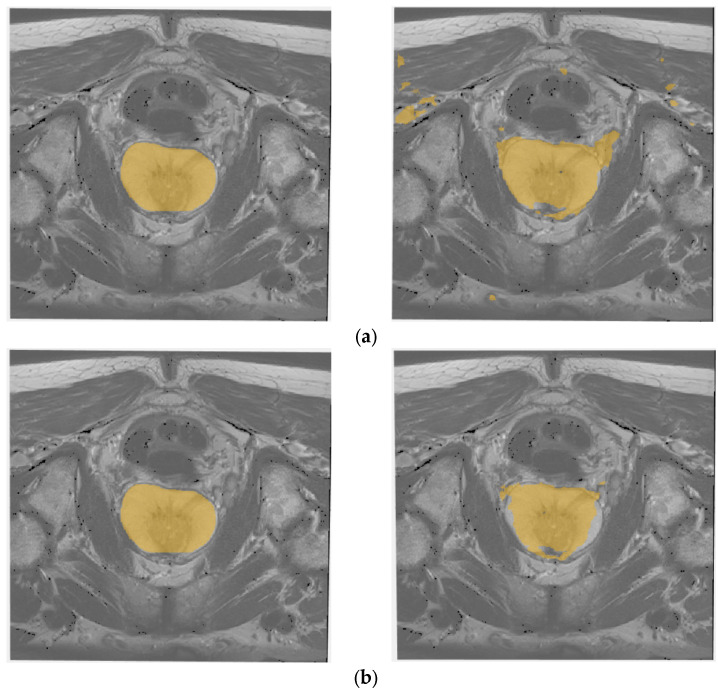
Visual comparison of prostate gland detection based on: (**a**) 3D U-Net (3), and (**b**) 3D AID-U-Net (2, 1). The slice images shown in the left column are the original CT-Scan slices accompanying with the pixel-wise annotations for prostate gland.

Finally, the visual results of instant segmentation of the prostate gland obtained by both 3D U-Net and AID-U-Net(2,1) are presented in Figure 10a,b.

### 4.6. Time Inference vs. Performance and Computational Complexity

**Figure 11:**
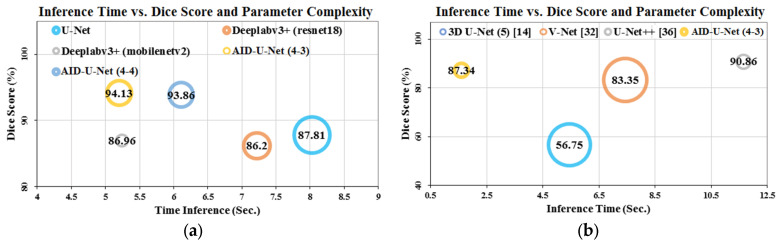
Time inference of several semantic segmentation networks vs. efficiency and different learnable parameters for processing of: (**a**) 2K optical colonoscopy test images belonging to Etis-Larib dataset, and (**b**) 2.4K CT-Scan volumes belonging to BraTS dataset based on the inference time calculations for the competitive methods proposed in [14] as 3D U-Net, [32] as V-Net, [36] as U-Net++ and Aid-U-Net with direct and sub-path combination as (4-3).

To complete the investigation on network segmentation performance in terms of inference time, computational complexity and efficiency, various architectures including U-Net, V-Net and U-Net++ along AID-U-Net were applied to 2D optical colonoscopy images of Etis-Larib dataset and 3D CT scan volumetric images of BraTs database. Figure 11 presents the comparison of inference time vs. dice score and computational complexity in terms of the number of learnable parameters. 

AID-U-Net features the shortest inference time for both 2D and 3D images, highest dice score for 2D images and lowest number of learnable parameters for 3D images. The superb performance of AID-U-Net can be contributed to its architectural design taking advantage of sub-contracting and sub-expansive paths. Furthermore, the cropping procedure as a pre-processing stage leads to lower redundant patches, and therefore, the performance of the network during the training and validation phases will be amplified.

## 5. Discussion and Conclusions

In the block diagram of a regular object extraction system, the presence of a segmentation block is inevitable since the boundaries and vicinity of the target objects should be determined for being deployed in the next processing steps such as object characterization and tracking. The proposed AID-U-Net provides an effective tool for accurate segmentation of the biomedical target objects in a shorter time and higher performance than the previously proposed U-Nets.

In this section, we first discussed the reasons why AID-U-Net with a specific (*K*,*d*) configuration outperforms another AID-U-Net with a different (*K*,*d*). In the last section of this part, a conclusion for the main topic of this paper was proposed. 

### 5.1. Discussion

The experimental results presented in the previous section show that our proposed AID-U-Net was able to achieve high quality results for semantic segmentation of target objects on several datasets. AID-U-Net also provides a semantic segmentation strategy with significant two-fold contributions. The contributions are low computational complexity and high dimensional flexibility. 

In this subsection, we discuss the differences between the performances of AID-U-Nets with different direct and sub-path depths. 

**Figure 12:**
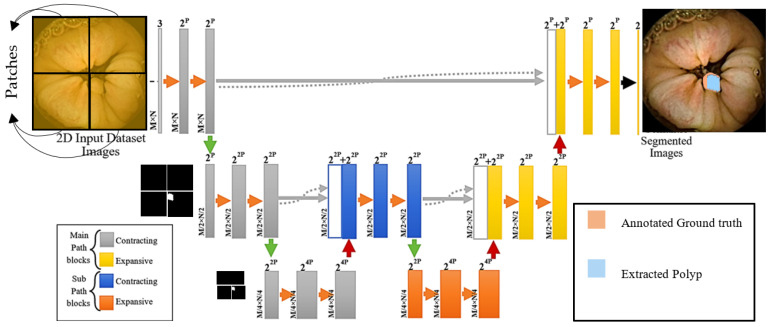
A visualization of polyp detection by means of a 2D AID-U-Net (2, 1). The final extracted polyp in the shadow of the original annotated ground truth is presented.

Generally, if the whole or a part of the target object inside a patch image is very small, then the high number of downsampling will lead to the disappearance of the whole or a part of the target object at the output of a deep CNN. For example, AID-U-Net (3, 1) outperforms all the other competitive networks including AID-U-Net (2, 2) for polyp detection in optical colonoscopy images, because the sizes of the input images are compatible with the cropping and patch extraction procedures. On the other hand, AID-U-Net (2, 2) outperforms all the other networks including AID-U-Net (3, 1) for CCE images. If the target objects (here are polyps in OC and CCE images) are small, there is a risk that in some patches and due to downsampling, some features will disappear. Given that, if the depth of sub-contracting and expansive paths is less than that of the layers that the non-zero ground truth exists for that object, the result of concatenation will include no part of the target object, and therefore, accuracy of the network will drop. Figure 12 illustrates these descriptions in a visual manner. 

The missed ground truth pixels belonging to the target object during the last downsampling layers of the direct contracting path cause an increase in the number of false negatives compared with an AID-U-Net with a smaller number of downsampling layers. In return, the AID-U-Net with a larger depth of direct path can reduce the number of false negatives, especially for images with initial sizes matched with the patch sizes and no need of cropping.

The strength of the proposed AID-U-Net is two-fold:

- The architecture provides a flexible deep neural network with lower computational complexity compared to the-state-of-the-art U-Nets.
- AID-U-Net can increase the accuracy of semantic segmentation of small and scattered target objects by deploying different lengths for the direct and sub-paths of this model.

Furthermore, the limitations are two-fold:

- First, the efficient combination of lengths for direct and sub-paths is not studied yet. As shown in [45], it is possible to embed pre-trained neural networks with specific lengths into the AID-U-Net. However, the lengths of direct and sub-paths for AID-U-Net architecture should be pre-defined, so that it could accept the pre-trained deep neural networks as its backbone.
- Acceptable range of the lengths for sub-paths has a maximum value *K*, limiting its functionality.

### 5.2. Conclusions and Future Work

The implementation and comparative results of this study show that by extracting and combining local features with respect to the global characteristics of the input image using sub-contracting and expansive paths, a significant improvement in the performance of the network for semantic segmentation tasks could be expected. This is the main distinguishing characteristic of our proposed network compared to U-Net or other similar architectures with no pre-trained backbones. We showed that by adopting this strategy, improvements in accuracy, IoU measures and BF score could be achieved, while simultaneously, the network took advantage of a significantly lower number of learnable parameters. This was particularly evident in the scenarios where 3D volumetric images were the target of segmentation tasks.

We further showed that by featuring sub-contracting and sub-expansive paths compromising between the extracted local features during direct paths, the final segmentation artifacts were reduced, or in some cases removed. The experimental results demonstrated the higher efficiency and improved performance of our network compared to other state-of-the-art solutions. In most cases, the best performance metrics in terms of accuracy, IoU, BF score, inference time and number of learnable parameters were achieved using our network. We are validating the performance of our network on two external databases, i.e., a 2D colon capsule endoscopy image featuring investigations of 2015 patients, and a 3D radiology image of prostate tumors collected on 1000 patients. This paves the way toward the deployment of our network in clinical practice for tasks related to the semantic segmentation of medical images.

Based on the experimental results, our main contributions are as follows:

1. Our proposed AID-U-Net model with two specific depth configurations for direct and sub-paths as (3, 1) and (2, 2), presented an average F_1_-Score increment of 3.82% for all 2D test data compared to that of a conventional U-Net and U-Net++.
2. Similarly, regarding the mean BF-Score, we observed an improvement of 2.99% for all 3D test data compared to that of V-Net and 3D U-Net.
3. The computational complexity of the proposed AID-U-Net model was significantly lower than the other competitive U-Nets, since the presence of sub-paths combined with the direct paths improves the low-scale object detection ability of the network and reduces the number of learnable parameters with the same number of layers in a conventional U-Net.

Besides all the benefits established by the proposed AID-U-Net, there would be some limitations. One of these limitations is that the depths for direct and sub-paths that can lead to the best performance for each dataset are found empirically, and there is no strict method for finding out the most efficient depths. The other limitation is related to the pre-trained weights and the strategies for mapping them with the proposed deep convolutional network. Given that, the future works would aim at overcoming the mentioned limitations. For example, one of the future works could aim at establishing a suitable strategy for finding the proper depth of direct and sub-paths, so that the best performance can be achieved. Furthermore, proposing a new strategy for adapting AID-U-Net to the deployment of a pre-trained backbone, without adding to the complexity of the model and reducing its flexibility, is another direction of research.

## Figures and Tables

**Table 1:** A summary of deep learning semantic segmentation methods for medical applications.

Semantic Segmentation Methods	Method’s Descriptions	Strengths	Limitations
U-Net [19]	FC layer modification	Applicable for low-volume training data. Mean IoU score of 92% for 2D and 87% for 3D dataset images.	Suffering from high hyperparameter complexity. Missing low-level features.
Circle-U-Net [20]	Circle-connectlayers, as the backbone of ResUNet-a architecture	Exceeds the Conventional U-Net on several standards. Captures context and expands paths more efficiently than U-Net.	Lack of flexibility for different contracting and circle-connect layers. Lower accuracy in the presence of gated signal attention (GSA).
Fully Convolutional DenseNet [22]	Feed-forward FCN with 2 Transitions Down (TD) and 2 Transitions Up (TU)	Outperforming upsampling paths from dense networks. Produce state-of-the-art results on standard benchmarks. Achieve a global accuracy of 88% on the CamVid dataset.	High computational complexity. Low accuracy for biomedical image segmentation.
DeepLabv3+ [23]	Convolutions with upsampling filters, Atrous Convolution, and CRFs	Segmenting objects at multiple scales by means of ASPP. Improving localization of object boundaries by using DCNNs.	Fine-tuning parameters. Supporting Atrous convolution and CRFs only for xception and mobilenetv2.
FastFCN [24]	Joint Pyramid Upsampling (JPU).	Achieving mIoU of 53.13% in PASCAL and 55.84 in ADE20K datasets. Running 3 times faster than FCN.	Unbalance flexibility between contracting and expansive paths.
W-Net [26]	A Deep Model for Fully Unsupervised Image Segmentation.	Achieving impressive segmentation results by concatenating two fully convolutional networks together into a single autoencoder.	High computational complexity. Targeting only unsupervised segmentation. Having a small portion of flexibility.
U-Net++ [34]	An efficient ensemble of U-Nets of varying depths with redesigning with Nested and dense skip connection.	Outperforming baseline models. Enhancing segmentation quality of varying-size objects. Fast and flexible.	Depending on skip connection and therefore, higher complexity. Not full-scale deep supervision.
U-Net3+ [35]	A full-scale connected U-Net with full-scale skip connections	Full-scale skip connections and deep supervisions. Reducing the network parameters to improve the computation efficiency.	High computational load due to hybrid loss function. Over-segmentation in non-organ images.
SD-UNet [36]	Stripping down U-Net for segmentation of Biomedical Images.	Small model size. Fewer parameters than U-Net. Fast inference time with a computational complexity.	1. Adapted to work on devices with low computational budgets.

**Table 2:** Database introduction.

Dataset	No. of Images	Input Size	Modality	Provider
CCE	4144	512 × 512	RGB Images	[4]
CVC-ClinicDB	612	288 × 384	RGB Images	[39]
CVC-ColonDB	379	574 × 500	RGB Images	[40]
CVC-ETIS-Larib	196	966 × 1225	RGB Images	[41]
G-A cells	230	696 × 520	Gray-level images	[42]
Brain Tumor	484	M × N × P	CT-Scan Voxels	[43]
Prostate Cancer	484	M × N × P	CT-Scan Voxels	[44]

**Table 3:** Comparative Results between U-Net with depth 4, AID-U-Net (3, 1) and AID-U-Net (2, 2), applied to optical colonoscopy images of CVC-ClinicDB for colorectal polyp segmentation.

Evaluation Metrics	U-Net++	U-Net (4)	AID-U-Net (3, 1)	AID-U-Net (2, 2)
F_1_-Score (%) (↑)	90.32	73.68	**91.00**	74.08
IoU (%) (↑)	82.34	58.33	**83.49**	58.83
Learnable Param.s ^1^(↓)	9.0 M	4.0 M	3.4 M	924 K

^1^ The learnable param.s factor *N* = 5 and the depth of convolutional activation for each layer 2*^N^* = 2^5^.

**Table 4:** Comparative results between U-Net with depth 4, AID-U-Net (3, 1) and AID-U-Net (2, 2) applied to images of colon capsule endoscopy (CCE) for colorectal polyp segmentation.

Evaluation Metrics	U-Net++	U-Net (4)	AID-U-Net (3, 1)	AID-U-Net (2, 2)
F_1_-Score (%) (↑)	87.64	81.19	86.82	**88.12**
IoU (%) (↑)	78.00	68.34	76.71	**78.76**
Learnable Param.s ^1^(↓)	9.0 M	4.0 M	3.4 M	**924 K**

^1^ The learnable param.s factor *N* = 5 and the depth of convolutional activation for each layer 2*^N^* = 2^5^.

**Table 5:** Comparative results between U-Net with depth 4, AID-U-Net (3, 1) and AID-U-Net (2, 2) applied to microscopic image segmentation.

Evaluation Metrics	U-Net++	U-Net (4)	AID-U-Net (3, 1)	AID-U-Net (2, 2)
F_1_-Score (%) (↑)	94.36	93.22	95.66	**98.18**
IoU (%) (↑)	89.32	87.30	91.68	**96.43**
Learnable Param.s ^1^(↓)	9.0 M	4.0 M	3.4 M	**924 K**

^1^ The learnable param.s factor *N* = 5 and the depth of convolutional activation for each layer 2*^N^* = 2^5^.

**Table 6:** Parameter configuration for 3D implementation of U-Net and AID-U-Nets.

Network-Related Parameters	U-Net	AID-U-Net(3, 1)	AID-U-Net(2, 2)
Direct contract Depth	4	3	2
Downsampling Coeff.	5	5	5
Sub-contract Depth	N/A	1	1
Total No. Layers	69	69	69
Learnable parameters ^1^	22.4 M	9.7 M	**3.1 M**

^1^ The number of learnable parameters is calculated based on factor *N* = 5. The depth of convolutional activation for each layer is determined based on a power of 2*^N^* = 2^5^, and it will increase with a power of 2 at each contracting step as shown in Figure 3b for the 3D models.

**Table 7:** Comparison between semantic segmentation results achieved by U-Net(3) and proposed AID-U-Net(2,1) for 3D brain tumor detection.

3D Network	Global Accuracy (%)	Mean Accuracy (%)	Mean IoU (%)	Weighted IoU (%)	Mean BF Score (%)
U-Net (3)	99.4	84.34	83.92	98.81	77.51
AID-U-Net (2, 1)	**99.67**	**92.83**	**91.35**	**99.35**	**89.19**

**Table 8:** Comparison between semantic segmentation results achieved by U-Net(3) and proposed AID-U-Net(2,1) for 3D prostate detection.

3D Network	Global Accuracy (%)	Mean Accuracy (%)	Mean IoU (%)	Weighted IoU (%)	Mean BF Score (%)
U-Net (3)	98.1	61.08	59.98	96.26	80.24
AID-U-Net (2, 1)	**98.99**	**93.54**	**83.23**	**98.21**	**93.27**

**Table 9:** Comparative results between semantic segmentation of 3D brain tumors achieved by 3D U-Net, V-Net and proposed AID-U-Net (3, 2).

3D Network	No. Layers	No. Learnable Parameters (↓)	Global Acc. (%) (↑)	Mean Acc. (%) (↑)	Mean IoU (%) (↑)	Weighted IoU (%) (↑)	Mean BF Score (%) (↑)
U-Net (5)	85	77.2 M	98.1	**98.24**	68.74	40.19	56.57
V-Net (5)	116	80.8 M	**99.62**	87.04	85.54	71.81	83.35
AID-U-Net (3, 2)	85	**10.9 M**	98.84	96.37	**88.15**	**78.43**	**87.34**

## Data Availability

The data supporting the results of capsule endoscopy investigations reported in this article can be provided to interested readers, by contacting the corresponding author. However, due to the absence of consent for publication or complete anonymization of the outcome of the clinical trial, enquiries about access to the outcome of the trial, i.e., database containing patient information and images of lesions should be made to the data owner, i.e., Odense Univesity Horpital (Svendborg Hospital). CVC-ClinicDB is available online at: https://polyp.grand-challenge.org/CVCClinicDB. Cell Tracking Challenge 2D Datasets is available online at: http://celltrackingchallenge.net/datasets. CVC-ColonDB is available online at: http://mv.cvc.uab.es/projects/colon-qa/cvccolondb. EtisLarib is available online at: https://polyp.grand-challenge.org/EtisLarib (accessed on 22 October 2022).
